# Predicting brace holiday eligibility in juvenile idiopathic scoliosis

**DOI:** 10.1007/s43390-024-00924-w

**Published:** 2024-07-08

**Authors:** Julianna Lee, Nathan Chaclas, Lucas Hauth, David VanEenenaam, Vineet Desai, John M. Flynn

**Affiliations:** 1 Department of Orthopaedics, The Children’s Hospital of Philadelphia, 3500 Civic Center Blvd., Philadelphia, PA 19104 USA; 2 Department of Orthopaedic Surgery, The University of Pennsylvania, Philadelphia, PA USA

**Keywords:** Idiopathic scoliosis, Bracing treatment, Brace holiday, Scoliosis brace

## Abstract

**Purpose:**

The psychological effects of scoliosis bracing can be difficult, and thus clinicians sometimes recommend a brace holiday when the curve corrects to less than 25°. However, the clinical indications for taking a break from the brace before reaching maturity have yet to be described. We hypothesized there would be a relationship between brace holiday eligibility and degree of curve at presentation, change in curve magnitude while bracing, and level of bracing compliance.

**Methods:**

A retrospective cohort study at a single institution was performed from 2016 to 2022. Objective brace compliance I-button data were collected on patients aged 3–9 years old. Patients with other etiologies besides idiopathic scoliosis before the age of 10 were excluded. Binary logistic regression was performed to determine the effect of significant variables on the likelihood of brace holiday.

**Results:**

Fifty-six patients met inclusion criteria. Of these, 20 were able to get a brace holiday. Patients with higher brace compliance and larger in-brace curve correction were more likely to get a brace holiday (*P* = 0.015, 0.004). Patients with higher BMIs and larger curves at initial presentation were less likely to get a brace holiday (*P* = 0.002, 0.014).

**Conclusion:**

Compliant brace wearers with good in-brace correction are most likely to be eligible for a brace holiday. While some elements remain immutable, others are modifiable, such as bracing compliance. Understanding how outcomes differ between patients who do and do not take a brace holiday will be crucial to elucidating if the psychological benefit of taking a break from the brace can be justified.

## Introduction

Idiopathic scoliosis makes up 80% of scoliosis cases and can develop at any time during childhood and adolescence. Because its natural history changes based on time of onset, this deformity is often classified by the age at which it develops: infantile (0–2 years), juvenile (3–9 years—JIS), adolescent (10–17 years—AIS), and adult (18 years and older) [1]. For curves between 25 and 50 degrees, management with orthotic bracing is the first line of treatment, with the primary goal being to prevent curve progression to surgical range (≥ 50°) until skeletal maturity has been reached [1]. Bracing has demonstrated success and can obviate the need for surgery; however, results to date show varying success rates [2–4].

JIS patients are at higher risk for more severe deformity because of the earlier age of onset as compared to AIS, which also necessitates a longer duration of bracing treatment. The psychological and social effects of bracing can sometimes be difficult and affect treatment compliance. Previous work has indicated that fear of peer rejection is central to many patients’ attitudes toward bracing [5]. Further studies in this area have linked bracing to detrimental views on physical appearance and have reinforced bracing as a tremendous psychosocial stressor in an impressionable group of patients that can negatively impact emotional response to stress, while heightening neuroticism and introversion [6, 7]. Bracing has further specifically been linked to increased risk for depression and anxiety in female patients, who make up the vast majority of this population [8]. Due to this, clinicians sometimes recommend a brace holiday [9, 10]. A brace holiday is a novel concept in orthopedics characterized by the discontinuation of bracing before skeletal maturity. It is indicated if the child has outgrown the brace and their curve has corrected down to below the bracing threshold of a 25-degree coronal Cobb angle and has remained there for at least 6 months [11]. Similar studies in the pediatric spine deformity literature have described the concept of a ‘cast holiday’ with respect to Mehta casting for early onset scoliosis, wherein a minimum of a 4 week break was offered to select patients [12].

While the Scoliosis Research Society has reached a consensus on bracing indications and duration, there remains significant variability in their use protocols throughout treatment duration [13]. Therefore, the present study aims to investigate the factors that determine brace holiday eligibility.

## Methods

A retrospective review of the electronic medical record was performed for children diagnosed with juvenile idiopathic scoliosis (JIS) prescribed a first-time, custom thoracic-lumbar-sacral orthosis brace from 2016 through 2022 at a single institution. Patients with initiation of bracing after 11 years of age, non-idiopathic etiologies (neuromuscular, syndromic, or congenital), loss to follow-up, and those who were still bracing were excluded.

Demographic data recorded included age, race, ethnicity, and BMI at brace prescription. All patients in this study were prescribed a Boston brace. Primary curve location and curve severity at time of brace prescription were recorded. After the brace was constructed, patients returned for an in-brace radiograph to measure in-brace correction. At the time of brace initiation, all patients were recommended full-time bracing protocol. Patients were followed every 4 to 6 months to assess curve progression. Bracing protocol and recommended hours at follow-up appointments were based on curve severity with a shared decision-making model.

Bracing hours were recorded based on temperature-sensitive compliance monitors collected at follow-up visits in the orthopedic clinic and/or visits to the orthotist. Compliance was measured as a percent compliance of hours worn out of hours prescribed. Hours prescribed were standardized to three denominators, 17 h, 14 h, or 8 h, based on prescribed protocol as either full-time, part-time, or night-time only. Average bracing compliance was calculated by averaging the percent compliance datapoints available for the duration of bracing.

Patients who received a brace holiday were followed with the plan to re-initiate bracing if the curve progressed back above the 25° threshold for bracing. For these patients, date of brace discontinuation and coronal Cobb angle of the primary curve were recorded at time of discontinuation. If bracing was re-initiated, coronal Cobb angle and date of new brace prescription were recorded.

Descriptive statistics were performed for all outcome variables. *T* tests and Mann–Whitney *U* tests were performed for continuous variables guided by normality analysis. Fisher exact testing was performed for categorical variables. Significant univariate analyses were incorporated into a binary logistic regression.

## Results

Fifty-six patients (fifty-two female) met inclusion criteria. The median age at brace prescription was 9.0 (4.9–10.9) years with a median BMI of 15.9 (12.9–26.4) kg/m2. The median primary curve was 29 (19–58) degrees. The mean in-brace correction was 58.2 ± 23.2%. The average brace compliance was: 83.9% (22–120%). Thirty patients (52%) were bracing for more than 13 h.

**Fig. 1:**
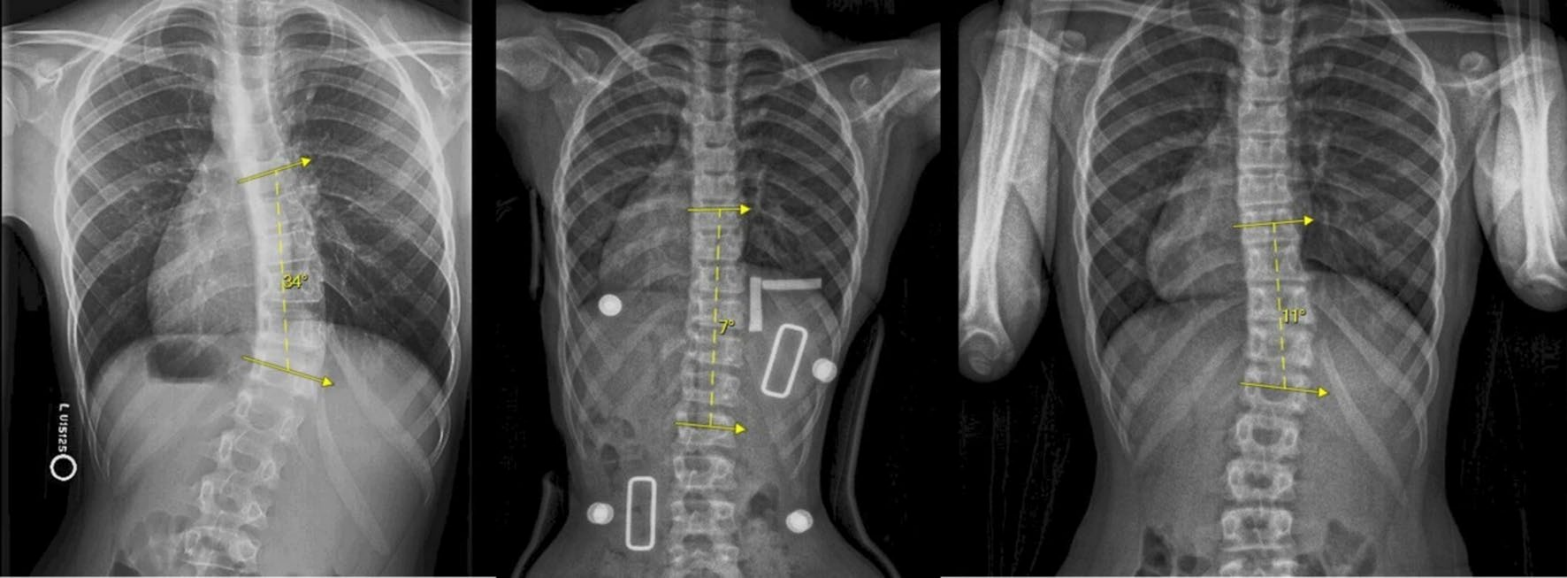
A patient with a 34-degree primary thoracolumbar curve at brace initiation (left) which corrected to 7 degrees in brace (center). The deformity was 11 degrees out of brace at follow-up with 105% brace compliance (right) and the patient was granted a brace holiday

Twenty patients (nineteen female) received a brace holiday. The median age at brace prescription was 8.1 years (5.1–10.7) with a median BMI of 14.9 kg/m2 (12.9–21.2). The median primary curve was 27.5° (20–34). The mean in-brace correction was 72.2 ± 22.8%. The mean brace compliance was 92.4 ± 19.1%. Fifteen (72%) of patients who took a brace holiday were bracing for more than 13 h. Of those that took a brace holiday, the mean bracing duration was 2.6 ± 0.9 years. They were able to discontinue bracing with a curve of 13.5 ± 5.5 degrees. An example patient is shown in Fig. 1. Of those that re-initiated bracing (*N* = 9), the average duration of brace holiday was 11.5 months (6–35). Patients re-initiated bracing at an average curve of 30.4° ± 4.0.

Thirty-six patients (thirty-three female) did not receive a brace holiday. The median age at brace prescription was 9.6 (5.0–10.9) years with a median BMI of 17.4 (13.4–26.4) kg/m2. The median primary curve was 32° (19–58). The median in-brace correction was 52.6%. (21.7–100) The mean brace compliance was 74% ± 26%. Fifteen (42%) of patients who did not get a brace holiday were bracing for more than 13 h. There were no significant differences in sex between the cohorts (*P* = 0.529). Patients with higher brace compliance (*P* = 0.015) and larger in-brace curve correction (*P* = 0.004) were more likely to get a brace holiday. Patients with higher BMIs (*P* = 0.002) and larger curves at initial presentation (*P* = 0.014) were less likely to get a brace holiday. Results are further summarized in Table 1.

**Table 1:** Demographics of Holiday and No-Holiday patient cohorts

	Holiday (*N* = 20)	No Holiday (*N* = 36)	Overall (*N* = 56)	*P* value
Sex				
Male	1	3	4	0.529 F
Female	19	33	52	
Age at brace prescription (years)	8.1 [5.1–10.7]	9.6 [5.0–10.9]	9.0 [4.9–10.9]	0.054 U
BMI (kg/m2)	14.9 [12.9–21.1]	17.4 [13.5–26.4]	15.9 [12.9–26.4]	0.002 U
Primary curve (deg)	27.5 [20–34]	32 [19–58]	29 [19–58]	0.014 U
In-brace correction (%)	72.2 ± 22.8	51.5 ± 20.5	58 ± 23.2	0.004 T
Compliance (%)	92.4 [55–120]	74.0 [22–112]	83.9 [22–120]	0.015 U
Sanders score at final brace discontinuation	5.7 ± 1.7	5.5 ± 1.7	5.6 ± 1.7	0.488 U

*U* Mann–Whitney *U* test, *T* two sample independent *T* test, *F* Fisher’s exact test

A binary logistic regression was performed to determine the effects of BMI, curve severity, in-brace correction, and brace compliance on the likelihood that a patient would receive a brace holiday. Increasing BMI (*P* = 0.019) and larger curves (*P* = 0.035) were associated with a decreased likelihood of being able to discontinue bracing, but better in-brace correction (*P* = 0.009) was associated with an increase in likelihood of being able to discontinue bracing. Results are further summarized in Table 2. Holiday sub-cohort stratification is described in Table 3.

**Table 2:** Logistic regression results for statistically significant demographic factors

	Std. error	*P* value	Exp(B)	95% CI
BMI	0.223	0.019	0.594	0.380–0.927
Primary curve (deg)	0.068	0.035	0.866	0.756–0.992
In-brace correction (%)	0.023	0.009	1.062	1.01–1.11
Compliance (%)	2.410	0.156	30.524	.246–3785.753

**Table 3:** Stratification of holiday sub-cohort

	Holiday ended (returned to bracing) (*N* = 10)	Holiday ongoing (*N* = 10)	Overall (*N* = 20)
Sex			
Male	1	0	1
Female	9	10	19
Age at brace prescription (years)	8.4 [5.1–10.2]	7.9 [5.7–10.7]	8.1 [5.1–10.7]
BMI (kg/m2)	15 [13.5–17.5]	15 [12.9–21.1]	14.9 [12.9–21.1]
Primary curve (deg)	29 [25–34]	26 [20–34]	27.5 [20–34]
In-brace correction (%)	66 ± 18	77 ± 26	72.2 ± 22.8
Compliance (%)	105 [80–120]	83 [55–108]	92.4 [55–120]
Pre-holiday bracing duration (years)	2.4 ± 0.7	2.7 ± 1.2	2.6 ± 0.9
Curve at holiday initiation (deg)	15.6 ± 3.9	11.6 ± 6.2	13.5 ± 5.5
Sanders score at first brace discontinuation	2.0 ± 0.8	2.5 ± 0.8	2.3 ± 0.8
Most recent sanders score	N/A	3.4 ± 0.5	N/A
Holiday duration (months)	11.5 [6–35]	N/A	N/A
Curve at return to bracing (deg)	30.4 [22–35]	N/A	N/A

## Discussion

Findings from this study strengthen the limited literature base for young scoliosis brace wearers with JIS who are offered a holiday when their deformity corrects after a period of treatment. Study cohort composition was in keeping with prior published literature on demographic factors and patient presentation. Bracing compliance monitoring in this study yielded a wide range of values, demonstrating significant variability in daily bracing hours among the study cohort. In addition to bracing compliance, primary curve magnitude, BMI, and in-brace correction were all associated with likelihood of receiving a brace holiday.

Previous literature has reported a mean age at brace prescription of 7.9 years old for JIS patients, with a primary curve of magnitude 61° [14]. Patients have often been reported to correct significantly in the brace (58 ± 24%) [4]. Similarly, our cohort consisted primarily of skeletally immature females with primary curves above the bracing threshold who corrected well in the brace.

Less has been reported with respect to patients who are granted a brace holiday. When patients received a holiday, their curves had corrected to about half the size they had been at presentation. These patients had significantly lower BMIs, higher compliance, higher in-brace correction, and smaller primary curves than their counterparts. While not statistically significant, our brace holiday sub-cohort was also braced earlier. Their holidays lasted over a year on average, with half of them returning to bracing during that time. When brace holiday patients returned to bracing, their curves were of comparable size to their original primary presenting curve (30.4 vs 27.5 degrees). With respect to guidance on initiation and termination of a brace holiday, the authors have considered offering holidays on a case-by-case basis in patients whose curves correct to under the 25-degree coronal Cobb angle threshold for bracing and demonstrate that they can stay under this threshold for 6 months (at two separate visits). These discussions are always framed under the shared decision-making model. These patients are virtually all pre-pubescent at this stage and are followed closely (about once every 6 months) as they near and enter puberty. Patients and families are counseled that the accelerated growth experienced during puberty can cause their curve to increase back up to above the 25-degree Cobb angle threshold, at which point a discussion on returning to bracing would be initiated.

Limitations of this study are due to its retrospective nature. While the negative psychosocial effects of scoliosis bracewear have been well documented in literature, work is still needed to quantify the benefit of a ‘brace holiday’ in concrete terms with objective patient-reported outcomes data. This would serve as important evidence distinguishing a perceived benefit from a verified benefit in these patients. Variables such as compliance were significantly different between the sub-cohorts on univariate analysis but were not significant on regression analysis. In addition, the size of the sub-cohort who initiated a brace holiday and then returned to bracing limited robust analysis. Lastly, temperature-sensitive monitors are not always completely accurate in recording daily wear hours, and temperature settings must sometimes be adjusted over time to ensure accurate reporting. Future work should, therefore, investigate further predictors of holiday eligibility in larger cohorts to better inform and guide patients and families, as well as attempt to quantify the extent of the psychosocial benefit these holidays have on our patients. Moreover, outcomes here must be evaluated to determine the safety of a brace holiday with respect to risk for curve progression and future surgery.

Our logistic regression analysis revealed that degree of in-brace deformity correction and BMI are the variables most predictive of a brace holiday, with factors such as brace compliance and primary curve size also playing a significant role. While BMI was statistically different between the Holiday and No-Holiday groups (*P* = 0.002), the difference between the two groups mean BMI’s (14.9 vs. 17.4 kg/m^2) is far less clinically significant compared to the difference between 92.4 and 74.0% brace compliance (the observed difference between the Holiday and No-Holiday groups, respectively) (*P* = 0.015). Therefore, we concluded from this data that in-brace correction and brace compliance are the most important and modifiable factors which can lead to a brace holiday.

In summary, this paper is useful to the pediatric spine clinician as it demonstrates that compliant brace wearers with significant in-brace deformity correction may be eligible for a brace holiday during their course of treatment. Additional work is necessary to better understand the factors that influence brace holiday duration. While some elements remain immutable, such as primary curve size, others are modifiable by the patient, such as bracing compliance. Understanding how outcomes differ between patients who do and do not get a brace holiday will be crucial to elucidating if the psychological benefit of taking a break from the brace can be justified. Ultimately, this study lends credence to the notion that patients can play an instrumental role in shaping their healthcare journey.

## Data Availability

The dataset used for this study is available upon request.

## References

[1] Negrini S, Donzelli S, Aulisa AG et al (2018) 2016 SOSORT guidelines: orthopaedic and rehabilitation treatment of idiopathic scoliosis during growth. Scoliosis Spinal Disord 13:3 29435499 10.1186/s13013-017-0145-8 PMC5795289

[2] Sponseller PD (2011) Bracing for adolescent idiopathic scoliosis in practice today. J Pediatr Orthop 31(1 Suppl):S53-60 21173620 10.1097/BPO.0b013e3181f73e87

[3] Babaee T, Kamyab M, Ganjavian MS, Rouhani N, Jarvis J (2020) Success rate of brace treatment for juvenile-onset idiopathic scoliosis up to skeletal maturity. Int J Spine Surg 14(5):824–831 33097584 10.14444/7117 PMC7671458

[4] Aulisa AG, Guzzanti V, Marzetti E, Giordano M, Falciglia F, Aulisa L (2014) Brace treatment in juvenile idiopathic scoliosis: a prospective study in accordance with the SRS criteria for bracing studies - SOSORT award 2013 winner. Scoliosis 9(1):3 24817906 10.1186/1748-7161-9-3 PMC4016774

[5] Gornitzky AL, England P, Kiani SN, Yellin JL, Flynn JM (2023) Why don’t adolescents wear their brace? A prospective study investigating psychosocial characteristics that predict scoliosis brace wear. J Pediatr Orthop 43(1):51–60 36194756 10.1097/BPO.0000000000002272

[6] Cheung MC, Law D, Yip J, Cheung JPY (2022) Adolescents’ experience during brace treatment for scoliosis: a qualitative study. Int J Environ Res Public Health 19(17):10585. 10.3390/ijerph191710585 36078297 10.3390/ijerph191710585 PMC9517878

[7] Matsunaga S, Sakou T, Nozoe S (1997) Psychological effects of brace therapy on patients with idiopathic scoliosis. J Orthop Sci 2(6):391–395

[8] Bae BH, Ham CH, Patel U, Suh Y (2023) Psychosocial effect of brace treatment in adolescent idiopathic scoliosis: a study using EQ-5D. Clin Spine Surg 36(10):E488 37482631 10.1097/BSD.0000000000001489

[9] Chan SL, Cheung KM, Luk KD, Wong KW, Wong MS (2014) A correlation study between in-brace correction, compliance to spinal orthosis and health-related quality of life of patients with adolescent idiopathic scoliosis. Scoliosis 9(1):1 24559234 10.1186/1748-7161-9-1 PMC3996075

[10] Rivett L, Rothberg A, Stewart A, Berkowitz R (2009) The relationship between quality of life and compliance to a brace protocol in adolescents with idiopathic scoliosis: a comparative study. BMC Musculoskelet Disord 10:5 19144157 10.1186/1471-2474-10-5 PMC2635344

[11] Lenke LG, Dobbs MB (2007) Management of juvenile idiopathic scoliosis. J Bone Joint Surg Am 89(Suppl 1):55–63 17272423 10.2106/JBJS.F.00644

[12] Fedorak GT, Dreksler H, MacWilliams BA, D’Astous JL (2020) What is the cost of a “cast holiday” in treating children with early onset scoliosis (EOS) with elongation derotation flexion (EDF, “Mehta”) casting? J Pediatr Orthop 40(8):396–400 32118800 10.1097/BPO.0000000000001533

[13] Halsey M, Dolan LA, Hostin RA et al (2021) Scoliosis research society survey: brace management in adolescent idiopathic scoliosis. Spine Deform 9(3):697–702 33580371 10.1007/s43390-020-00265-4

[14] Whitaker AT, Hresko MT, Miller PE et al (2022) Bracing for juvenile idiopathic scoliosis: retrospective review from bracing to skeletal maturity. Spine Deform 10(6):1349–1358 35852786 10.1007/s43390-022-00544-2 PMC9579105

